# Health-related behaviors during adolescence and subsequent anxiety and depression: the HUNT study

**DOI:** 10.1093/eurpub/ckac131.482

**Published:** 2022-10-25

**Authors:** AL Kleppang, MV Vettore, I Hartz, SH Haugland, TH Stea

**Affiliations:** 1 Department of Health and Nursing Sciences, University of Agder, Kristiansand, Norway; 2 Innlandet Hospital Trust, Innlandet, Norway; 3 Department of Psychososial Health, University of Agder, Kristiansand, Norway

## Abstract

**Background:**

Evidence on the predictors of common mental disorders using nationwide health registries are scarce in Norway. Identifying modifiable behaviours affecting mental health across the lifespan is paramount to develop tailored strategies to tackle mental illnesses. The aim of this study was to identify patterns of health-related behaviours in adolescence and their influence on anxiety and/or depression in adulthood.

**Methods:**

This was a prospective study based on data from the Trøndelag Health Study (HUNT) and health register data (N = 2061). Patterns of health-related behaviours were assessed according to physical activity, consumption of wholegrain bread, fish, fruit, vegetables, and sugar sweetened beverages and insomnia. Participant’s use of healthcare system for anxiety and/or depression was recorded at least once in the health registries. The patterns of health-related behaviors were identified through latent class analysis. Multivariable logistic regression was used to test the association between patterns of health-related behaviors and depression or/and anxiety.

**Results:**

Four classes of health-related behaviors were identified: class 1 (15.2%), class 2 (36.0%), class 3 (24.2%), class 4 (24.6%). Adolescents with unhealthy behaviors (classes 1, 2 and 3) had 82%, 34% and 84% higher odds of depression and/or anxiety during adolescence and early adulthood than those from the healthy-related behaviors group (class 4).

**Conclusions:**

Our findings suggest that health-related behaviors are clustered among Norwegian adolescents. There was a meaningful association of the three patterns of unhealthy behaviors during adolescence with anxiety and depression in adulthood. Population strategies and policies aiming to tackle unhealthy behaviors among adolescents can positively impact on adult’s mental health.

**Key messages:**

• Improving healthy behaviors during adolescence may reduce the burden of mental illness in adulthood.

• Population strategies and policies aiming to tackle unhealthy behaviors among adolescents can positively impact on adult’s mental health.

